# The effect of hospital volume on patient outcomes in severe acute pancreatitis

**DOI:** 10.1186/1471-230X-12-112

**Published:** 2012-08-17

**Authors:** Hsiu-Nien Shen, Chin-Li Lu, Chung-Yi Li

**Affiliations:** 1 Department of Intensive Care Medicine, Chi Mei Medical Center, No. 901 Chung-Hwa Road, Yong-Kang City, Tainan, Taiwan; 2 Department of Medical Research, Chi Mei Medical Center, No. 901 Chung-Hwa Road, Yong-Kang City, Tainan, Taiwan; 3 Department of Public Health, College of Medicine, National Cheng Kung University, Tainan, Taiwan; 4 Department of Public Health, College of Public Health, China Medical University, Taichung, Taiwan

**Keywords:** Severe acute pancreatitis, Hospital volume, Outcomes

## Abstract

**Background:**

We investigated the relation between hospital volume and outcome in patients with severe acute pancreatitis (SAP). The determination is important because patient outcome may be improved through volume-based selective referral.

**Methods:**

In this cohort study, we analyzed 22,551 SAP patients in 2,208 hospital-years (between 2000 and 2009) from Taiwan’s National Health Insurance Research Database. Primary outcome was hospital mortality. Secondary outcomes were hospital length of stay and charges. Hospital SAP volume was measured both as categorical and as continuous variables (per one case increase each hospital-year). The effect was assessed using multivariable logistic regression models with generalized estimating equations accounting for hospital clustering effect. Adjusted covariates included patient and hospital characteristics (model 1), and additional treatment variables (model 2).

**Results:**

Irrespective of the measurements, increasing hospital volume was associated with reduced risk of hospital mortality after adjusting the patient and hospital characteristics (adjusted odds ratio [OR] 0.995, 95% confidence interval [CI] 0.993-0.998 for per one case increase). The patients treated in the highest volume quartile (≥14 cases per hospital-year) had 42% lower risk of hospital mortality than those in the lowest volume quartile (1 case per hospital-year) after adjusting the patient and hospital characteristics (adjusted OR 0.58, 95% CI 0.40-0.83). However, an inverse relation between volume and hospital stay or hospital charges was observed only when the volume was analyzed as a categorical variable. After adjusting the treatment covariates, the volume effect on hospital mortality disappeared regardless of the volume measures.

**Conclusions:**

These findings support the use of volume-based selective referral for patients with SAP and suggest that differences in levels or processes of care among hospitals may have contributed to the volume effect.

## Background

The performance of a hospital is linked to the hospital volume of various surgical procedures and medical conditions [1-6]. Generally, high-volume hospitals have shown to be more efficient and have better outcomes than low-volume hospitals. Documentation of the volume-outcome relationship is important because patient outcomes may be improved through volume-based selective referral [1,3].

An inverse volume-outcome relationship was revealed in patients with acute pancreatitis (AP) [5,6]. However, prior studies suffered from shortcomings that may have overestimated the volume effect and limited the generalization of findings [5-7]. These shortcomings included failures to exclude readmissions or recurrences [5,6], consider the severity of AP [5], account for the hospital clustering effect [6], and model hospital volume as a continuous variable [5,6,8,9]. Besides, the definition of high volume hospitals considerably differed between the studies (≥118 cases/year *vs.* >16 cases/9 months) [5,6], which limited the practical application of the results. Moreover, the inclusion of both mild and severe cases in these studies [5,6] implied that the results were generalized to all AP patients, which was unreasonable because selective referral was not justified for mild and self-limited diseases, such as mild AP [10,11]. Therefore, we conducted this study and enrolled only severe cases from a national cohort of patients with first-attack AP [12] to investigate the effect of hospital volume on patient outcomes.

## Methods

### Database

Data regarding the patients were obtained from the Taiwan National Health Insurance Research Database (NHIRD), which is released for research purposes by the National Health Research Institute and is one of the largest and most comprehensive databases in the world [12,13]. Information included in the inpatient database included sex, date of birth, encrypted patient identification numbers, residential or work area, dates of admission and discharge, medical institutions providing services, the International Classification of Diseases, Ninth Revision, Clinical Modification (ICD-9-CM) codes of diagnoses (up to five) and procedures (up to five), outcome at hospital discharge (recovered, died or transferred), order codes and hospital charges. The study was exempt from obtaining approval from the Human Subjects Institutional Review Board and informed consent from the patients due to the use of an encrypted administrative database.

### Definitions and patients

**Figure 1:**
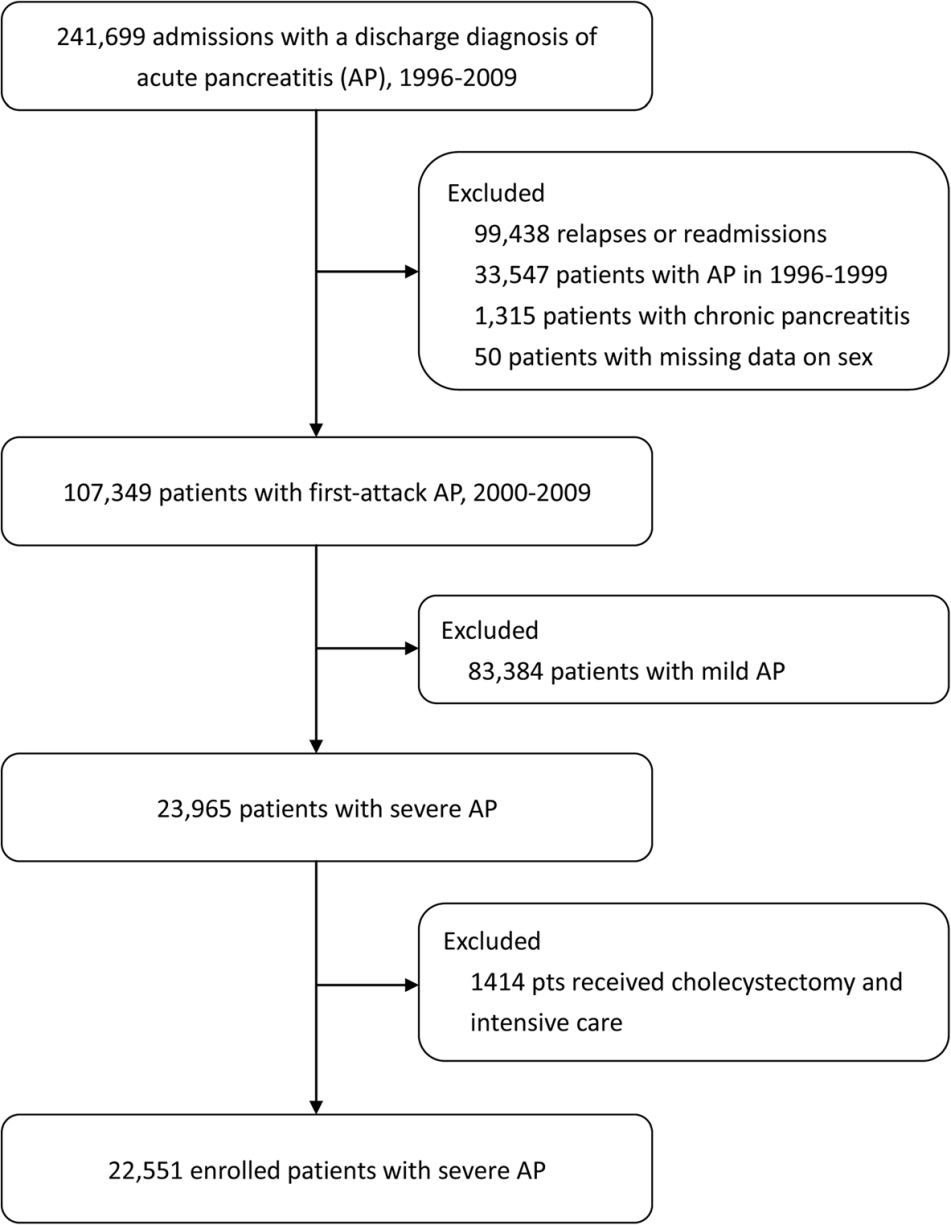
**Study flow diagram.** (Note: Patients hospitalized for AP between 1996 and 1999 were excluded to ensure the inclusion of first-attack cases because most relapses occur within the first 4 years after the first "attack)."

AP was defined by ICD-9-CM code 577.0 in any position of the five diagnoses [12]. Severe AP (SAP) was defined primarily according to the Atlanta classification scheme [14], but was modified by the presence of intensive care unit (ICU) admission (as a surrogate of acute physiology and chronic health evaluation [APACHE] II score ≥8), organ dysfunction or failure, gastrointestinal bleeding, or local complications [12,13]. The enrollment of the patients is shown in Figure 1. We excluded patients (*n* = 1,414) with biliary AP who received cholecystectomy and intensive care and had no organ failure, gastrointestinal bleeding, or local complications because these patients may have had mild biliary AP and received ICU care only after the surgery [12]. After exclusion, we enrolled 22,551 patients in the following analysis.

### Exposure variable

**Figure 2:**
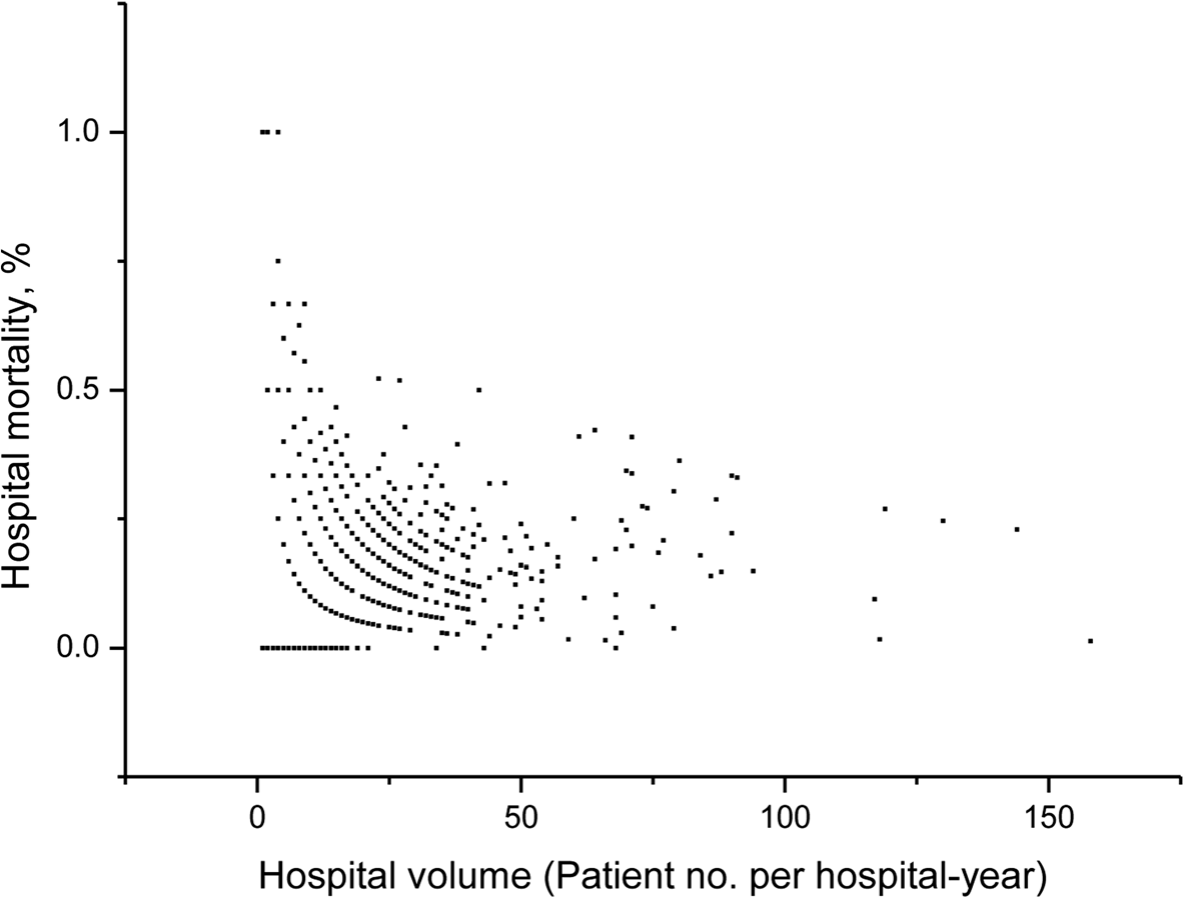
**Distribution of hospital volume *versus *hospital mortality per hospital-year in severe acute pancreatitis (Note: There were 467 hospitals contributing to a total of 2,208 hospital-years.** Median hospital volume was 5 cases per hospital-year [interquartile range 2 − 13]).

The annual number of SAP cases per hospital was the main exposure variable. The distribution of hospital volume *versus* hospital mortality per hospital-year is shown in Figure 2.

We first measured hospital volume as a continuous variable (per 1 case increase per hospital-year) to assess the effect of hospital volume on outcomes [8]. Then, the hospital volume was sorted and divided into 4 and 9 about-equal subsets, respectively, to help visualize the effect of increasing volume and for practical uses. The quartile ranges were 1, 2 − 5, 6 − 13 and ≥14 SAP cases per hospital-year, respectively. The 9-quantile ranges (corresponding proportion of hospital-year units) were 1 (22.0%), 2 (12.2%), 3 (8.2%), 4-5 (10.4%), 6-8 (11.1%), 9-12 (10.1%), 13-19 (10.7%), 20-33 (9.3%), and ≥34 (5.9%) SAP cases per hospital-year, respectively. Patients were allocated essentially in decentile except that the first two decentiles with 1 case per hospital-year were collapsed into one subset due to the skewed nature of the volume. The quartiles were used for both presentation and comparison of the results and the 9-quantiles were used primarily to show the trend of volume effect on hospital mortality.

### Covariates

Two levels of covariates, baseline and additional, were included. The baseline covariates were patient and hospital characteristics. The patient characteristics included age, sex, year of admission, urbanization (urban, suburban and rural area) [15], Charlson comorbidity index [16,17], and causes (biliary, alcohol-related, both or others) and severity criteria of AP.

The hospital characteristics included hospital level (medical center [>500 beds], regional [250–500 beds] and district hospitals [20–249 beds]) [13], hospital ownership (public, private not-for-profit, or private for-profit) [18] and geographical location (northern, central, southern, and eastern Taiwan).

Additional covariates were employed to account for the process of care and included the following selected treatments: cholecystectomy and life-support measures (including total parenteral nutrition [TPN], hemodialysis, vasopressors and mechanical ventilation [MV]) [12].

### Outcomes

Primary outcome was hospital mortality [12]. Secondary outcomes were hospital charges and hospital length of stay (LOS). The charges were adjusted to the 2009 price levels in United States Dollars (USD) [12].

### Statistics

Data were analyzed using the SAS software, version 9.1 (SAS Institute, Inc., Cary, NC, USA). Continuous variables are presented as median (interquartile range, IQR); discrete ones as count or percentage. A two-tailed p value of <0.05 was considered significant.

We hypothesized that hospital volume is inversely associated with hospital LOS, charges and mortality. We assumed that the relationship is linear. In the univariate analysis, we performed ANOVA test for the linearity of scaled variables and linear-by-linear association Chi-square test for categorical data. To account for clustering, the effect of hospital volume was analyzed by using regression model with generalized estimating equations methods [19], specifying an exchangeable structure of a working correlation matrix, to construct regression models. Hospital mortality was regressed with a logit link function and hospital LOS and charges were log-transformed and then regressed with a linear link function on hospital volume. Both univariable and multivariable analyses were performed to yield the crude and adjusted estimates. In the multivariable analysis, we performed two consecutive models adjusting for the baseline covariates in model 1 and for the baseline and additional covariates in model 2. We examined the volume effect by first using hospital volume as a continuous variable and then as a categorical variable, as aforementioned. The effects of hospital volume are presented as odds ratios (OR) with 95% confidence interval (CI) for hospital mortality, and as percentage changes with 95% CI, which were calculated from the exponentiated regression coefficients minus 1 [20], for hospital LOS and charges. Model performance was assessed by using R-squared and c statistics. The variance of outcomes explained by hospital volume was assessed and compared with that of other covariates by using the coefficient of determination (*r*^*2*^) for hospital LOS and charges and, by the percentage change of -2 log likelihood (-2LL) for hospital mortality. The change in -2LL (%) was calculated by dividing the difference in -2LL values between the univariable and the intercept-only models by the corresponding value of the intercept-only model. The *r*^*2*^ was derived from the univariable linear regression model. We examined the estimated slope coefficients and the standard errors of the mean and found no indication of collinearity.

## Results

### Hospital and patient characteristics

Table 1 shows the characteristics of the SAP patients. Hospital volume was correlated with hospital level, ownership and geographic location. Low quartile hospitals tended to be at the district level, private-for-profit ownership, and located in southern Taiwan, whereas higher quartile ones tended to be at the regional or center level, public or private not-for-profit ownership and located in the north.

**Table 1:** **Characteristics of hospitals and patients with severe acute pancreatitis (*n*** **= 22,551)**

**Variables**	**Hospital volume quartiles (by case No. per hospital-year)**				**P values for trend**
	**1**	**2-5**	**6-13**	**14+**	
**Hospital characteristics**					
** No. of hospital-years**	486	679	514	529	−
** No. of hospitals**	276	261	165	104	−
** Hospital level, %**					<0.001
** Medical center**	0	0.5	5.0	39.1	
** Regional hospital**	4.5	16.8	46.7	48.1	
** District hospital**	95.5	82.7	48.3	12.8	
** Hospital ownership, %**					<0.001
** Private (for-profit)**	70.3	53.2	32.9	17.8	
Private (not-for-profit)^*^	10.8	17.6	33.9	50.7	
** Public**	18.9	29.2	33.2	31.5	
** Geographic location, %**					<0.001
** Northern**	24.5	36.2	39.6	37.4	
** Central**	29.8	26.5	28.4	28.3	
** Southern**	39.7	32.4	25.1	29.5	
** Eastern**	6.0	4.9	6.9	4.7	
**Patient No. by year of admission**					<0.001
** 2000-2001**	129	578	929	2150	
** 2002-2003**	102	410	1037	2689	
** 2004-2005**	95	400	953	3520	
** 2006-2007**	89	379	873	3544	
** 2008-2009**	71	338	803	3462	
**Patient characteristics**					
** Median age (IQR), yr**	50 (38-70)	52 (39-72)	54 (41-73)	57 (42-73)	<0.001
** Male sex, %**	75.1	72.4	68.0	64.9	<0.001
** Urbanization, %**					0.039
** Urban**	45.8	49.8	49.9	50.8	
** Suburban**	37.7	34.6	35.7	34.6	
** Rural**	16.6	15.6	14.3	14.6	
** Charlson Comorbid Index, %**					0.688
** 0**	15.6	20.6	21.1	24.9	
** 1**	47.1	42.9	42.8	37.0	
** 2**	24.9	23.4	20.8	21.5	
** ≥3**	12.3	13.1	15.3	16.5	
**Causes, %**					<0.001
** Biliary stone**	10.3	15.0	16.6	17.1	
** Alcohol abuse**	4.1	3.5	4.0	4.1	
**Severity criteria**					
** ICU admission**	24.5	37.1	45.4	49.7	<0.001
** Organ failure**	48.4	51.6	52.2	60.1	<0.001
** Gastrointestinal bleeding**	42.2	37.5	33.2	27.5	<0.001
** Local complications**	3.7	4.2	7.1	7.0	<0.001
**Treatments, %**					
** Cholecystectomy**^†^	1.0	1.0	1.6	1.9	0.001
** Total parenteral nutrition**	1.9	2.7	6.7	12.7	<0.001
** Vasopressors**	14.8	18.2	21.3	26.1	<0.001
** Hemodialysis**	4.5	4.2	`7.4	9.7	<0.001
**Mechanical ventilation**	17.5	18.2	22.4	30.0	<0.001
**Median hospital LOS (IQR), d**	6 (4-13)	7 (3-13)	8 (4-16)	10 (5-20)	<0.001
**Median hospital charges (IQR), USD**	515 (284-1736)	713 (354-1845)	1069 (510-2814)	1715 (713-4619)	0.044
**Hospital mortality, %**	12.8	12.2	14.2	16.6	<0.001

IQR: interquartile range; ICU: intensive care unit; LOS: length of stay; USD: United States dollars.

^*^ A private not-for-profit hospital is a tax-exempt, commercial and entrepreneurial organization, which operates roughly in the same way with a private for-profit hospital except the difference in missions and goals on providing community benefit services [18].

^†^The surgery was performed during admission for first-attack acute pancreatitis.

More than two-thirds of the patients were treated in the highest quartile hospitals, and the proportion increased over time. With increasing hospital volume, patients tended to be older and less male-predominance, to live in urban areas and to have more complex comorbidities (Charlson-Comorbidity Index ≥3) and biliary causes. Among severity criteria of AP, organ failure was the most common, but the distributional pattern of the individual severity criteria differed. As hospital volume increased, the prevalence fell in gastrointestinal bleeding, but increased in ICU admission, organ failure and local complication.

More patients received TPN, vasopressors, hemodialysis, and MV in higher volume hospitals. Cholecystectomy was rarely performed in SAP and tended to be done in higher volume hospitals.

### Hospital volume-outcome relationship

The crude estimates showed that median hospital LOS and charges increased with hospital volume. A similar trend was observed for hospital mortality except that the mortality slightly fell in the second volume quartile (Table 1).

When hospital volume was entered into the regression models as a continuous variable (Table 2), the unadjusted effect of volume on hospital mortality was not statistically significant. Model 1 shows that hospital volume was inversely associated with hospital mortality. After controlling for additional treatment-related covariates, the volume effect on hospital mortality attenuated and became insignificant (Model 2). Volume, as a continuous variable, appeared to have no effect on hospital LOS, but was inversely associated with lower hospital charges in the unadjusted and fully adjusted models.

**Table 2:** **Effects of hospital volume (as a continuous variable) on outcomes in patients with severe acute pancreatitis (*n*** **= 22,551)**

**Outcomes**	**Crude OR or percent change (95% CI)**	**Adjusted OR or percent change (95% CI)**	
		Model 1^*^	**Model 2**^**†**^
**Hospital LOS**	−0.08% (-0.28%, 0.12%)	−0.06% (-0.25%, 0.13%)	−0.10% (-0.26%, 0.05%)
*R-squared*	*--*	*22.74%*	*30.31%*
**Hospital charges**	−0.17% (-0.32%, -0.01%)	−0.14% (-0.33%, 0.06%)	−0.16% (-0.27%, -0.04%)
*R-squared*	*--*	*53.92%*	*64.16%*
**Hospital mortality**	0.999 (0.994, 1.003)	0.995 (0.993, 0.998)	0.999 (0.996, 1.002)
*c statistic*	*--*	*85.4%*	*93.0%*

OR: odds ratio; CI: confidence interval; LOS: length of stay.

^*^ Covariates in model 1 included age (as a continuous variable), sex, year of admission, Charlson-Comorbidity Index (categorized as 0,1,2 and ≥3), urbanization, hospital level, the ownership of hospital, the region of hospital, causes of acute pancreatitis (categorized into biliary, alcohol-related, both or others), intensive care unit admission, organ failure, gastrointestinal bleeding and local complication.

^†^ Model 2 enrolled all covariates of model 1 and additional treatment covariates, including cholecystectomy, total parenteral nutrition, vasopressors, hemodialysis and mechanical ventilation.

**Figure 3:**
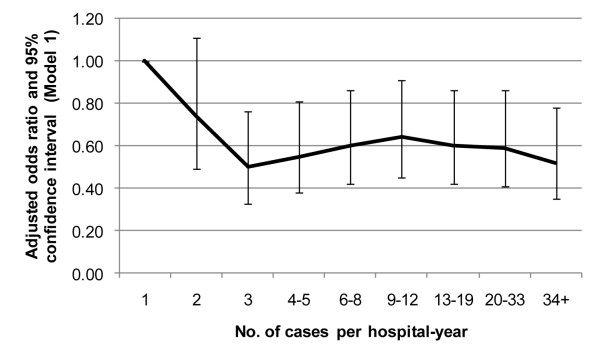
Effect of hospital volume on hospital mortality in severe acute pancreatitis adjusting for patient and hospital characteristics (Note: Hospital volume was divided into 9 about-equal subsets).

When hospital volume was entered into the regression models as a categorical variable (Table 3), the results of the volume effect on hospital mortality were similar to those modeled using volume as a continuous variable. Compared to the lowest quartile hospitals, SAP patients treated in higher volume hospitals had nearly 40% less risk of hospital mortality (Model 1). Volume effect became insignificant when differences in treatment among volume quartiles were controlled. When hospital volume was divided into 9 about-equal subsets, the effect of volume on hospital mortality appeared to plateau at ≥3 SAP cases per year (Figure 3). However, the results of hospital LOS and charges differed from those using volume as a continuous variable. For example, compared to the lowest quartile hospitals, patients treated in higher volume hospitals had a shorter hospital stay after controlling for the baseline covariates (Model 1). The volume effect on hospital LOS attenuated but persisted after additional adjustment of the treatment covariates (Model 2). Conversely, the volume effect on hospital charges was significant only after controlling for the baseline covariates (Model 1).

**Table 3:** **Effects of hospital volume (as a categorical variable) on outcomes in patients with severe acute pancreatitis (*n*** **= 22,551)**

**Outcomes**	**Hospital volume**	**Crude OR or percent change (95% CI)**	**Adjusted OR or percent change (95% CI)**	
			Model 1^*^	**Model 2**^**†**^
**Hospital LOS**				
	**Quartile 1**	Ref.	Ref.	Ref.
	**Quartile 2**	−2.52% (-12.78%, 8.94%)	−15.64% (-24.13%, -6.21%)	−12.95% (-21.22%, -3.82%)
	**Quartile 3**	0.00% (-11.15%,12.56%)	−16.78% (-25.57%, -7.02%)	−14.95% (-23.46%, -5.50%)
	**Quartile 4**	0.00% (-4.50%, 23.62%)	−16.36% (-25.05%, -6.05%)	−14.80% (-23.84%, -4.70%)
	*P for trend*	*0.909*	*0.005*	*0.007*
	*R-squared*	*--*	*23.81%*	*30.36%*
**Hospital charges**				
	**Quartile 1**	Ref.	Ref.	Ref.
	**Quartile 2**	8.65% (-4.50%, 23.62%)	−9.99% (-18.65%, -0.41%)	−5.15% (-13.38%, 3.86%)
	**Quartile 3**	7.39% (-7.63%, 24.85%)	−10.03% (-19.18%, 0.14%)	−5.60% (-14.28%, 3.96%)
	**Quartile 4**	9.25% (-6.79%, 28.05%)	−11.42% (-20.90%, -0.80%)	−6.79% (-15.92%, 3.33%)
	*P for trend*	*0.398*	*0.053*	*0.205*
	*R-squared*	*--*	*53.89%*	*64.16%*
**Hospital mortality**				
	**Quartile 1**	Ref.	Ref.	Ref.
	**Quartile 2**	0.98 (0.73, 1.31)	0.58 (0.42, 0.82)	0.75 (0.49, 1.15)
	**Quartile 3**	1.09 (0.83, 1.45)	0.62 (0.45, 0.88)	0.83 (0.54, 1.26)
	**Quartile 4**	1.02 (0.77, 1.35)	0.58 (0.40, 0.83)	0.75 (0.49, 1.16)
	*P for trend*	*0.720*	*0.012*	*0.281*
	*c statistic*	*--*	*85.4%*	*93.0%*

OR: odds ratio; CI: confidence interval; LOS: length of stay.

^*^ Covariates in model 1 included age (as a continuous variable), sex, year of admission, Charlson-Comorbidity Index (categorized as 0,1,2 and ≥3), urbanization, hospital level, the ownership of hospital, the region of hospital, causes of acute pancreatitis (categorized into biliary, alcohol-related, both or others), intensive care unit admission, organ failure, gastrointestinal bleeding and local complication.

^†^ Model 2 enrolled all covariates of model 1 and additional treatment covariates, including cholecystectomy, total parenteral nutrition, vasopressors, hemodialysis and mechanical ventilation.

**Figure 4:**
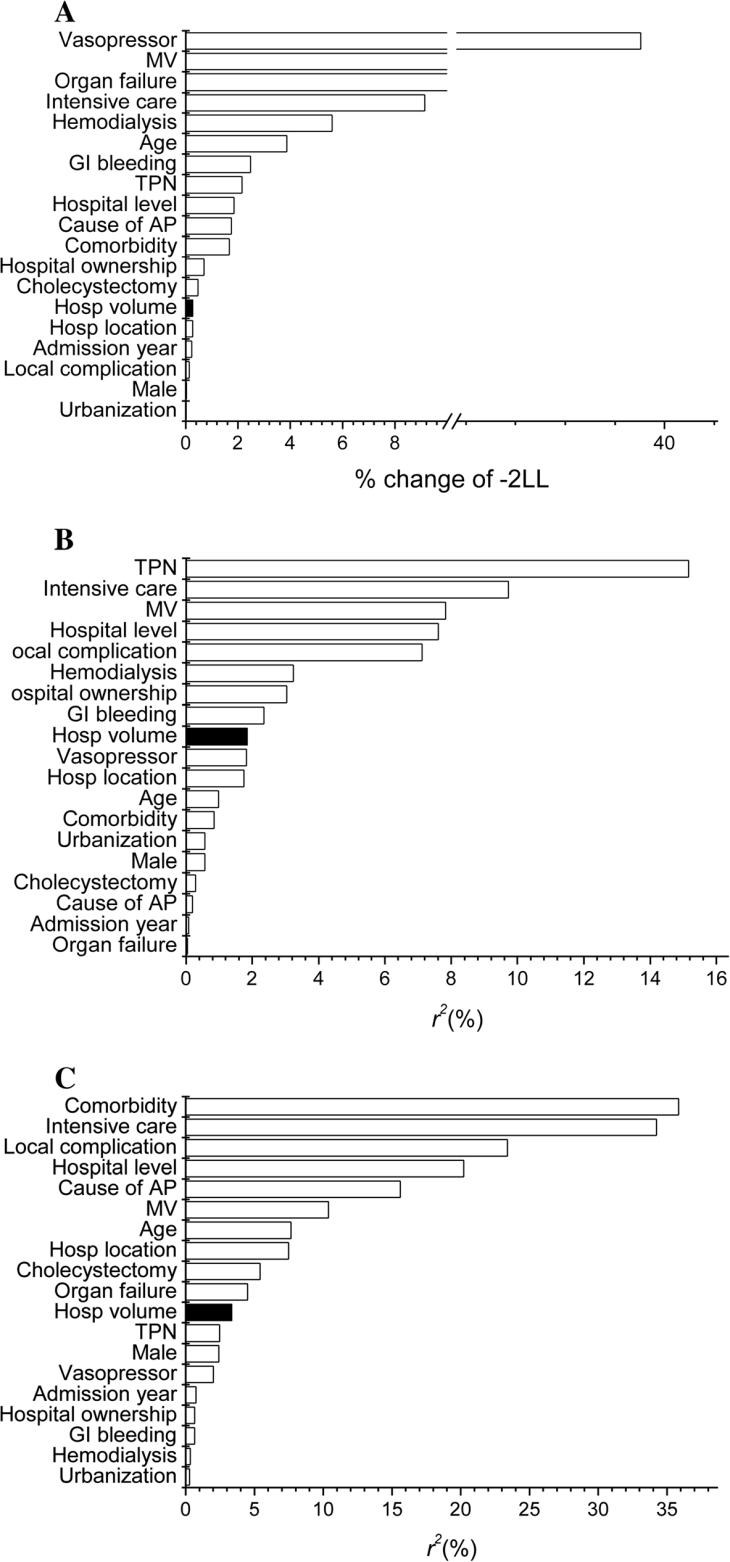
Variances of outcomes (A: hospital mortality, B: hospital length of stay, C: hospital charges) explained by various variables (MV: mechanical ventilation; TPN: total parenteral nutrition; AP: acute pancreatitis).

The variances in the outcomes explained by various variables are shown in Figure 4. The extent of the effect of hospital volume was greater on hospital mortality than on hospital LOS and charges. Nevertheless, the degree of the effect associated with hospital volume on various outcomes was relatively small compared to those of most patient and hospital characteristics.

## Discussion

In this study, we found that hospital SAP volume, either as a continuous or as a categorical variable, was inversely associated with hospital mortality after controlling for the baseline covariates. The volume effect disappeared when the differences in treatment among hospitals were controlled, suggesting that the effect can be largely explained by the different levels or processes of care across hospitals. Although the results on hospital LOS and charges were somewhat dependent on the definition of volume measures, trend analyses suggested that higher hospital volume appeared to be associated with shorter stay and lower cost.

We found that SAP patients treated in higher volume hospitals had nearly 40% less risk of hospital mortality than the lowest volume quartile ones. A recent report from Japan showed an even greater risk reduction in high volume hospitals [6]. In the report, Murata and coworkers [6] analyzed 7,007 patients with AP, including 662 severe cases, in 776 hospitals recruited over a 9-month period and found that in-hospital mortality (within 30 days) was about 66% lower for severe cases in high volume hospitals (defined as >16 AP cases) than in low volume ones (<10 AP cases). Had they limited their analysis to the volume of SAP cases and extended the study period to one year, the cutoff point for the lowest volume category (about 1 SAP case/year) would be very close to our results. However, in addition to working with a relatively small sample size, they also failed to exclude readmissions or recurrences of AP, and did not account for the hospital clustering effect in the analysis. Therefore, the effects are likely to be overinflated [21]. Besides, they defined SAP only as a dichotomous variable, which may have limited severity adjustment. Moreover, similar to an earlier study in the United States [5], they included both mild and severe cases in the analysis, which implied that volume-based selective referral, if adopted, would be applied to all patients with AP. However, some potential disadvantages of the volume-based policy [11] make us believe that selective referral should be limited to high risk patients, such as SAP cases. The transfer to a distant high-volume hospital is unreasonable for a mild AP patient who would recover within several days without the need of specific treatment other than simple supportive care [10].

The beneficial effects associated with high hospital SAP volume are likely related to the overall experience of the health care team, the processes of care and some organizational features, which included the availability of specialists, interventions and intensive care in complicated cases [5,11,22]. We found that the volume effect in SAP patients disappeared after additional adjustment of cholecystectomy and life-support measures, indicating that different levels or processes of care may have contributed to the observed effect. This finding is interesting and novel because previous studies did not consider the variation of treatment regimens across hospitals [5,6], which may be a probable mechanism for the observed relationship between volume and outcome. High volume hospitals are likely to have more specialists, interventions, and advanced intensive cares that are critical in saving the lives of pancreatitis patients. Moreover, we found that hospital mortality plateaued at ≥3 cases/year, suggesting a possible threshold effect of hospital volume on SAP outcome. The threshold effect may also be responsible for the insignificant association between hospital volume, as continuous variable, and hospital LOS and charges. Because the number of cases per year was within the margin of error of other values (Figure 3), the threshold value requires further validation.

This present study has important implications on the health policy and future research for the treatment of pancreatitis in Taiwan. Our recent study shows that hospital charges per patient with acute pancreatitis in Taiwan increased by nearly 50% from 2000 to 2009 [12]. Most of the increase was likely due to the lack of a formal referral system and an improper reimbursement policy. In Taiwan, reimbursement for some services (e.g., physician staffing and ICU bed) increases with hospital levels, which has promoted the growth of higher level hospitals out of proportion to lower level hospitals [23]. Consequently, patients with mild pancreatitis who could be treated properly in lower level hospitals usually sought medical care in higher level hospitals, leading to an overall higher cost of health care for pancreatitis. The reimbursement policy may also help explain why hospital volume did not affect hospital charges in this study because most hospitals with lower quartile volumes were lower level hospitals (i.e., district hospitals) and most of the hospitals with higher quartile volumes were higher level hospitals (i.e., regional hospitals or medical centers). The findings in the present study support the need for a better referral system that can limit the access of patients with mild pancreatitis and transfer severe cases to high-volume hospitals in Taiwan, which may lead to appropriate reallocation of medical resources. However, further research is needed to examine the outcome of transfer and the cost-effectiveness of the volume-based selective referral.

Some limitations deserve comments. First, the definition of SAP in this study tended to include patients who had a more severe attack and had received intensive care and/or invasive treatments. For example, some patients might not be included if they had an APACHE II score ≥8 but were cared for only outside an ICU or if they had local complications but did not receive invasive procedures. Besides, some patients with organ failure may also be missed because of the limited number of diagnostic codes. However, the selection of a more severe group of patients is non-differential among hospitals, which tends to bias the observed effect towards the null. Second, residual confounding may be present because adequate adjustment for potential confounders may be lacking, which is especially true for biliary and alcohol-related AP. The potential bias arising from residual confounding is uncertain. Finally, the generalizability of the findings may be limited by the different prevalence of causes in other regions of the world because the hospital volume-outcome relationship may be more important for biliary causes (e.g., expertise in ERCP) of pancreatitis as compared to alcoholic causes.

## Conclusions

The results of this study support the use of volume-based selective referral for patients with SAP. The volume threshold was rather low (i.e., 3 cases/year) and would not pose a significant caseload for current high volume hospitals. The outcomes of the transfer of SAP patients to high volume hospitals and its cost-effectiveness require further research.

## Abbreviations

-2LL: -2 log likelihood; AP: Acute pancreatitis; APACHE II score: Acute physiology and chronic health evaluation II score; CI: Confidence interval; ICD-9-CM: International classification of diseases ninth revision, clinical modification; ICU: Intensive care unit; IQR: interquartile range; LOS: Length of stay; MV: Mechanical ventilation; OR: Odds ratio; NHIRD: National health insurance research database; SAP: Severe acute pancreatitis; TPN: Total parenteral nutrition; USD: United States dollar.

## Competing interests

The authors declare that they have no competing interests.

## Authors’ contributions

HNS designed the study, obtained funding, performed data mining and processing, did statistical analyses, drafted the initial manuscript, and revised important content. CLL contributed to the study design, data mining and processing, analyses and interpretation of results, and revision for important content. CYL participated in the interpretation of results and revision for important content. All authors read and approved the final manuscript.

## Pre-publication history

The pre-publication history for this paper can be accessed here:

http://www.biomedcentral.com/1471-230X/12/112/prepub
